# SYSTEMATIC REVIEW OF THE LITERATURE ON SURGICAL TREATMENT OF CHRONIC RHINOSINUSITIS IN CHILDREN: WHAT IS THE BEST APPROACH?

**DOI:** 10.1590/1984-0462/2020/38/2018068

**Published:** 2020-01-13

**Authors:** Camila Taniguti Cordeiro Vasco, Heloisa Carvalho de Morais, Melissa Ameloti Gomes Avelino

**Affiliations:** a Hospital Alberto Rassi, Goiânia, GO, Brazil.

**Keywords:** Child, Surgery, Sinusitis, Chronic disease, Criança, Cirurgia, Sinusite, Doença crônica

## Abstract

**Objective:**

To carry out a systematic literature review on the surgical treatment of chronic rhinosinusitis in the pediatric population.

**Data sources:**

A bibliographic review methodology was used, based on data from National Library of Medicine (Medline), PubMed, Latin American and Caribbean Health Sciences Literature (LILACS) and Scientific Electronic Library Online (SciELO), of the indexed works from 2006 to 2016, including the pediatric population from zero to 13 years of age. The search keywords according to Medical Subject Heading (MESH) and Health Sciences Descriptors (DeCS) were: child, surgery, sinusitis and chronic disease. A total of 318 articles were collected, five of which met the inclusion criteria and were used as a basis for this review. All articles were prospective cohort studies, level of evidence 2B, according to the criterion used by evidence-based medicine.

**Data synthesis:**

The literature agreed that the next step for the cases refractory to drug treatment in chronic rhinosinusitis in childhood would be surgery. Adenoidectomy would be the initial method, for the safety of the procedure and improvement in about 50% of the cases, although more significant results were found in patients who associated this procedure with facial sinus surgery.

**Conclusions:**

Surgical treatment should be indicated for chronic rhinosinusitis in childhood after treatment failure. The results pointed out that adenoidectomy, when associated with some type of approach to the facial sinus, present better results.

## RESUMO

**Objetivo:**

Realizar uma revisão sistemática da literatura sobre o tratamento cirúrgico da rinossinusite crônica na população pediátrica.

**Fonte de dados:**

Utilizou-se metodologia de revisão bibliográfica, por meio de levantamentos nas bases de dados científicas National Library of Medicine (Medline), PubMed, Literatura Latino-americana e do Caribe em Ciências da Saúde (LILACS) e Scientific Electronic Library Online (SciELO), dos trabalhos indexados no período entre 2006 e 2016, incluindo a população pediátrica de zero a 13 anos. Os descritores de busca, segundo o Medical Subject Heading (MESH) e os Descritores em Ciências da Saúde (DeCS), foram: *Child, Surgery, Sinusitis e Chronic Disease*. Foram levantados 318 artigos, dos quais cinco preencheram os critérios de inclusão e foram usados como base para esta revisão. Todos os artigos foram estudos de coorte prospectivos, nível de evidência 2B, segundo critério usado pela medicina baseada em evidências.

**Síntese dos dados:**

Foi consenso na literatura que, para os casos refratários ao tratamento medicamentoso na rinossinusite crônica na infância, o próximo passo seria a cirurgia. A adenoidectomia seria o método inicial, pela segurança do procedimento e pela melhora em cerca de 50% dos casos, embora resultados mais significativos terem sido encontrados em pacientes que associaram esse procedimento à cirurgia dos seios da face.

**Conclusões:**

O tratamento cirúrgico pode ser indicado na rinossinusite crônica na infância após falha terapêutica. Os resultados apontaram que a adenoidectomia, quando associada a algum tipo de abordagem aos seios, apresenta melhores resultados.

## INTRODUCTION

Pediatric chronic rhinosinusitis (CRS) can be defined, according to the European Position Paper on Rhinosinusitis and Nasal Polyps 2012 (EPOS 2012), as the presence of two or more symptoms in which at least one is nasal obstruction/nasal congestion or nasal rhinorrhea (anterior or posterior). Sometimes it is accompanied by facial pain/ pressure and a cough for at least 12 weeks and a paranasal sinus computed tomography (PNS CT) or a nasal endoscopy is performed.^1^

Pediatric CRS is very common in clinical history and negatively affects quality of life and learning ability.^2,3^ The pathophysiology of pediatric CRS does not necessarily mirror that of the adult population, although heterogeneity and overlap exist in both populations. Factors that may contribute to pediatric CRS include ostial obstruction, recurrent upper respiratory infection (URI), allergy, immaturity or deficiency of the immune system, biofilm formation in the sinus and adenoid tissues, anatomical anomalies, gastroesophageal reflux (GERD), adenoid hypertrophy, and disorders that alter the mucociliary clearance.^4-6^

The mainstay of treatment is clinical, which includes antibiotic therapy coupled with topical, systemic corticosteroids and saline irrigation.^2^ Surgery is reserved for cases of drug treatment (TM) failure, where symptoms persist for a period of 12 weeks, and are associated with changes in computed tomography (CT), which is suggestive of CRS, according to the Lund-Mackay criteria.^7-9^ The persistence of nasosinusal symptoms in the pediatric population can also be assessed by the questionnaire Sinus and Nasal Quality of Life Survey (SN-5), which stratifies quality of life and correlates it with the CT findings.^9-10^

In the literature there are several studies on CRS that address surgical procedures. However, most of these studies exclude pediatric patients because they present prominent differences from the adult population. One of these differences is at birth, when paranasal sinuses are relatively undeveloped in comparison to those of an adult.^5^ During childhood, complete pneumatization and expansion of the sinuses occur with the development of the sphenoid and frontal sinuses at about age seven. Children also typically have hypertrophic adenoid tissue, which may play an obstructive role and serve as a reservoir for microorganisms, forming biofilms and maintaining CRS. ^5,6^

EPOS 2012 suggested that the surgical algorithm for pediatric CRS should begin with an adenoidectomy and that concomitant dilation of the maxillary sinus balloon or antral irrigation could be considered. Functional endoscopic sinus surgery (FESS) is reserved for failures in treatment, patients without adenoid hypertrophy or in patients with disorders that directly affect mucociliary function.^1^ However, there is no consensus on the surgical treatment for childhood CRS.^7^ This study aimed to perform a bibliographic review of the studies that evaluated the surgical treatment of CRS in children and tried to define the best approach.

## LITERATURE REVIEW

A systematic review study was conducted to evaluate the best surgical treatment for children with CRS. Prisma guidelines were followed for the systematic reviews.^11^ To organize the problem, we used the PICO approach, which stands for: *Patient* (0 to 13 years old patients), *Intervention* (surgical or drug treatment), *Comparison* (comparison between treatment types) and *Outcomes* (results). A search of the Latin American and Caribbean Health Sciences Literature (LILACS), the National Library of Medicine (MedLine), PubMed, and the Scientific Electronic Library Online (SciELO) databases was performed between April and July 2017, in English, Portuguese and Spanish. The descriptors were “Child”, “Surgery”, “Sinusitis” and “Chronic disease”, the AND interlocutor was used, and the search was performed according to the Medical Subject Heading (MeSH) and its Portuguese equivalents, established by the Health Sciences Descriptors (DeCS).

For the selected articles, the following inclusion criteria were used:

- Randomized controlled trials and prospective studies.
- Periodicals indexed from 2006 to 2016.
- Pediatric age of up to 13 years old.
- Therapeutic failure in previous clinical treatment for CRS; the patient may or may not have had an adenoidectomy.

Exclusion criteria were:

- Texts with insufficient data for the study.
- Journals found in more than one database (duplicates).
- Articles unrelated to surgical treatment.
- Predisposing factors for CRS such as cystic fibrosis, immunoglobulin deficiency, ciliary dysfunction, syndromic diseases and craniofacial abnormalities.
- Editorials, theses, indications, guidelines, reports and case series.

For data analysis, studies found with the distribution of articles by year, authors, type of study, objective and results for comparative evaluation were organized in a table. Because this was a data review of the literature, approval from the Research Ethics Committee (*Comitê de Ética em Pesquisa* - CEP) was not required. The studies that met the inclusion/ exclusion criteria were selected blindly and independently by two authors. In cases where there was disagreement between the first two authors, the opinion of a third author was used. Because there were not many studies that met the inclusion and exclusion criteria and they had different clinical outcomes, it was not possible to perform a meta-analysis for a quantitative evaluation. Therefore, we opted for performing only a qualitative analysis.

**Figure 1:**
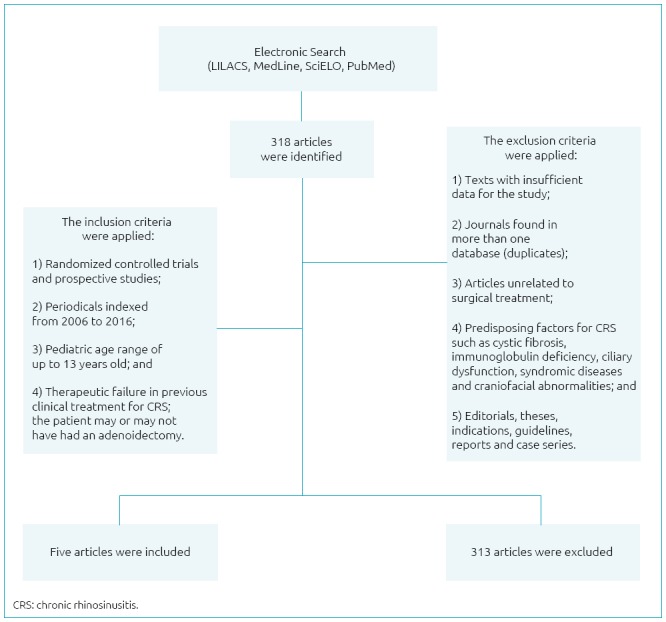
Flowchart of the methodology utilized in the study.

Initially, 318 studies were surveyed. After reviewing the titles and abstracts, and using the inclusion and exclusion criteria, five articles were selected for this review. The reference lists of these five studies were analyzed to verify the possibility of inclusion of new studies that also addressed the theme, but no other articles were added. The details of the selection process are summarized in Figure 1. Of the five articles selected, all of them were prospective cohort studies, but none of them were randomized. The main features are summarized in Table 1, and the results and conclusions of each study are summarized in Table 2.

**Table 1:** Characteristics of the included studies.

Author (year)	Age (years)	n (female/male)	Study Design
Ramadan et al. ^12^	3 to 13	60 32/28	Prospective
Ramadan et al. ^14^	4 to 11	49 27/22	Prospective
Ramadan et al. ^15^	4 to 12	26 6/20	Prospective
Wang et al. ^2^	7 to 12	79 37/42	Prospective
Seth et al. ^16^	6 to 12	35 8/27	Prospective

**Table 2:** Results and conclusions of the evaluated studies.

Author (year)	Key Results *	Conclusion
Ramadan et al.^12^	A + SL = 87.5% A = 60.7%	Children with a more severe sinus disease proven by the Lund-Mackay CT score had a higher success rate with maxillary sinus lavage at the same time as A. Children with a low CT score do not have this benefit.
Ramadan et al.^14^	B = 80% A = 52.6%	Balloon sinuplasty, in addition to being a safe procedure, was more effective than A alone, especially in older children.
Ramadan et al.^15^	B = 81%	Balloon dilation proved safe and effective in those patients in whom A failed.
Wang et al. ^2^	B = 92% TM = 44%	The balloon is a safe and effective method for the treatment of proven DT resistant pediatric CRS.
Seth et al. ^16^	FESS = 91.4%	In pediatric patients that are refractory to DT, FESS provides improvement in the symptom score and quality of life.

A: adenoidectomy; SL: sinus lavage; CT: computed tomography; B: balloon dilation of the sinuses; CRS: chronic rhinosinusitis; DT: drug treatment; FESS: functional endoscopic sinus surgery; * efficacy of procedures after 12 months of follow-up using the Lund-Mackay radiological criteria or the SN-5 quality of life scale.

## DISCUSSION

In this study, 249 children were evaluated, 148 were male and their ages ranged between three and 13 years old. All of the studies were prospective and none of them were randomized. The criterion used by almost all of the studies except for the first article, ^12^ was evaluating the success or failure of surgical treatment using the SN-5 scale.^9^ Results were based on the 12-month SN-5 score compared with the preoperative SN-5 score. SN-5 scores correlated with the PNS CT findings according to the Lund-Mackay scores.^10^ As originally described by Kay and Rosenfeld, a decrease of 0.5 to 1.0 on the SN-5 scale represented slight improvement; a decrease from 1.0 to 1.5 represented moderate change; and over 1.5 represented major improvement.^9^ Any case with a decrease in the SN-5 score ≥0.5 was considered a success.

The first article^12^ was the only one to not use the SN-5 scale as a therapeutic success/failure criterion. This study compared patients who underwent an adenoidectomy versus patients who underwent an adenoidectomy with a maxillary sinus irrigation. Sixty patients were evaluated who had failed clinical treatment for at least 26 weeks, which was documented by a PNS CT. Scores were presented according to the Lund-Mackay criteria.^13^ Exclusion criteria were patients who had previously undergone nasal surgery or an adenoidectomy as part of treatment. Both the surgeon and patients’ parents/ guardians chose which procedure would be performed. Patients were accompanied 1, 3, 6, 9, and 12 months after surgery. A questionnaire was administered 12 months after the procedure to assess preoperative symptoms and what had changed after the surgery. The symptoms evaluated included nasal obstruction/congestion, purulent drainage, a cough and headache. Parental satisfaction was also included. Children who needed another surgery were considered to be a failure. The group that underwent an adenoidectomy with maxillary sinus irrigation had an improvement of 87.5% when compared to the group that only underwent an adenoidectomy (60.7%). Children with a high SPN CT score (≥6) received more benefits in the hybrid procedure when compared to the adenoidectomy alone. However, when this association was extended to patients with low PNS CT scores (<6), there was no statistical difference between the groups.^12^ This article suggests that combined treatment has better results when the sinuses are involved.

Ramadan et al. ^14^ reaffirmed that an adenoidectomy is the first surgical treatment for CRS because of its technical ease and safety, but it is effective in 50% of patients and offers less benefits for those with asthma or those who are older than six years old. This study evaluated whether balloon dilation, with or without an adenoidectomy, was more effective than an adenoidectomy alone, and whether it could also be an option for treatment before FESS. Forty-nine children with clinical treatment failure who underwent surgical treatment were evaluated. Of these, 30 underwent balloon dilation sinuplasty which may or may not have been associated with an adenoidectomy. In the group that underwent a balloon sinuplasty, 80% of the patients had their symptoms improve after 12 months of the procedure, compared to the group that underwent an adenoidectomy alone (an improvement of 52.6%). The aim of the study was not to compare balloon dilation with FESS, but to assess whether balloon dilation could be an option in patients who would already be undergoing an adenoidectomy. The authors concluded that balloon dilation is not only an effective option, but also safe for the pediatric population. This article reinforces the previous statement, in which the success rates of the combined adenoidectomy sinuses procedure have better outcomes.

In another 2012 study, Ramadan et al. evaluated balloon dilation in patients who had already undergone an adenoidectomy for CRS treatment, and the treatment had failed. The SN-5 questionnaire was applied to 26 patients who met the inclusion criteria and were reevaluated 12 months after the procedure. The SN-5 scores improved significantly compared to preoperative values, and there was a total of 21 children (81%) who were successfully treated by balloon dilation. Patient age, gender and whether or not they had allergies or asthma were not significantly correlated. Although balloon dilation was safe and effective in patients with a failed adenoidectomy, four patients required a hybrid procedure (a maxillary antrostomy or an anterior ethmoidectomy). Therefore, in children with hypoplastic sinuses or older children with significant ethmoid disease, balloon sinuplasty may not be effective. However, in the absence of prospective controlled studies, the management of these children should be addressed individually.^15^ This article with children undergoing an adenoidectomy draws attention to cases of failure in patients that only receive an adenoidectomy. This justifies the combined choice of undergoing two procedures for patients that already have to go to surgery, as advocated by some surgeons.

Wang et al.^2^ analyzed sinus balloon dilation of the maxillary and frontal sinuses associated with saline irrigation that had dexamethasone and gentamicin. Only some patients had undergone an adenoidectomy. The researchers compared them to a control group using drug therapy. This study showed that balloon sinuplasty could significantly improve long-term symptoms and quality of life, according to SN-5 score and the Visual Analog Scale (VAS). In a separate analysis, the rate of improvement in patients with adenoid hypertrophy was 100%. In another analysis, which assessed only patients with pansinusitis associated with mucosal thickening in the frontal sinus, ethmoid, maxillary sinuses and sphenoids, balloon dilation in the frontal and maxillary sinuses alone was sufficient to control the symptoms.^2^ The study by Ramadan et al.^6^ had an improvement rate of 87.5% in patients with an adenoidectomy associated with sinus lavage (SL), similar to the study by Wang et al. (92%).^2^ The article itself suggests that irrigation during the procedure, and not necessarily balloon dilation, could explain the positive results. The balloon’s role could be to provide a proper channel to irrigate the sinuses and improve sinus drainage. This article reinforces the significant improvement of FESS in the surgical treatment of CRS in children.

The study performed by Seth et al.^16^ evaluated 35 children undergoing FESS after clinical treatment failure and demonstrated a statistical difference, with the improvement of symptoms and postoperative quality of life. The study also concluded that FESS is a safe procedure. Patients with grade 2 adenoid hypertrophy were excluded here.^17^ FESS consists of enlarging the natural ostia of the maxillary sinuses and ethmoids, preserving most or all of the sinus mucosa. It is emerging as a surgery option for CSR in children. When properly indicated, the results are good, with an expected improvement of 80 to 100%.^16^

Although there is a tendency for the surgical approach in CRS to start with an adenoidectomy in patients that are refractory to clinical treatment (because of the important role of chronic adenoiditis as a bacterial biofilm reservoir), the studies in this review showed better results when adenoidectomy was associated with some surgical approach to the sinuses (FESS, balloon sinuplasty or SL). However, no study emphasized which patient profile would benefit from an adenoidectomy alone or an adenoidectomy with a hybrid procedure. There is a tendency for better results from this association in patients with higher CT scores.^12^

Many articles in recent decades have emphasized the need for less invasive surgical procedures before submitting a pediatric patient to FESS. Among these, there is the use of balloon sinuplasty, saline instillation SL, dexamethasone or antibiotics and combined techniques. FESS has shown high success rates in the treatment of pediatric CRS, and previous concerns that it would have adverse consequences on facial development have proved to be unfounded.^4^ Balloon dilation has shown similar efficacy to FESS for refractory cases, and it is a safe strategy.^2^ Thus, although there is no consensus on the best surgical approach for the sinuses in children, surgical treatment has been considered more and more, and there is no future consequences for the development of these sinuses, as shown in this review.

Although all of articles showed favorable results, with significant improvement in long-term symptoms of patients undergoing surgery, most studies had limitations. Only five articles were found and none of them were randomized. Furthermore, a small number of children were evaluated. This failure may be justified because children rarely need surgical treatment for CRS.

The great difficulty and the existence of limited studies on childhood CRS are mainly due to the difficulty of establishing a confident diagnosis in this age group, especially in children under six years of age, since these children have recurrent URI, with three to eight episodes per year, while adults and adolescents have two to four episodes per year. These episodes may progress in about 2% of patients to acute bacterial rhinosinusitis and contribute to the development of CRS. Other factors involved in the pediatric population are immune system deficiency and the presence of biofilms in adenoid tissues, which tend to be resolved in adulthood. ^4^

Nasal polyposis is less common in the pediatric population with CRS than in the adult population, and when found, secondary diseases such as cystic fibrosis need to be investigated.^4^ However, a minority of pediatric CRS, about 18%, evolve into polypoid degeneration.^18^ The factors implicated in the evolution to a more severe disease are unknown, but may be associated with genetic factors, like the propensity to develop eosinophilic tissue reactions, and asthma. Other potential sequelae of pediatric CRS are chronic inflammatory bone changes, with bone thickening and maxillary sinus hypoplasia. The impact of these sequelae in adulthood is not yet clear. ^4^

Given the potential risk that pediatric CRS may develop into persistent, complicated CRS, early and more aggressive intervention is needed for the most severe cases, in order to avoid these long-term consequences. However, it should be kept in mind that such complications are present in only a minority of cases and therefore it is important to assess the severity of the disease and the impact on the child’s quality of life in order to make a surgical decision. Furthermore, the parents’ decision should be included.

## FINAL COMMENTS

CRS surgery in children is an option for treatment after appropriate drug therapy has failed. There are several approaches in the literature for the surgical treatment of paranasal sinuses. In this review, the best results were presented when adenoidectomy was associated with some type of sinus approach (SL, balloon sinuplasty or FESS).
